# Identification of GLPG/ABBV-2737, a Novel Class of Corrector, Which Exerts Functional Synergy With Other CFTR Modulators

**DOI:** 10.3389/fphar.2019.00514

**Published:** 2019-05-09

**Authors:** Gert de Wilde, Maarten Gees, Sara Musch, Katleen Verdonck, Mia Jans, Anne-Sophie Wesse, Ashvani K. Singh, Tzyh-Chang Hwang, Thierry Christophe, Mathieu Pizzonero, Steven Van der Plas, Nicolas Desroy, Marlon Cowart, Pieter Stouten, Luc Nelles, Katja Conrath

**Affiliations:** ^1^Galapagos NV, Mechelen, Belgium; ^2^AbbVie, North Chicago, IL, United States; ^3^Department of Medical Pharmacology and Physiology, University of Missouri, Columbia, MO, United States; ^4^Galapagos SASU, Paris, France

**Keywords:** cystic fibrosis transmembrane conductance regulator (CFTR), cystic fibrosis, chloride channel, electrophysiology, protein misfolding

## Abstract

The deletion of phenylalanine at position 508 (F508del) in cystic fibrosis transmembrane conductance regulator (CFTR) causes a severe defect in folding and trafficking of the chloride channel resulting in its absence at the plasma membrane of epithelial cells leading to cystic fibrosis. Progress in the understanding of the disease increased over the past decades and led to the awareness that combinations of mechanistically different CFTR modulators are required to obtain meaningful clinical benefit. Today, there remains an unmet need for identification and development of more effective CFTR modulator combinations to improve existing therapies for patients carrying the F508del mutation. Here, we describe the identification of a novel F508del corrector using functional assays. We provide experimental evidence that the clinical candidate GLPG/ABBV-2737 represents a novel class of corrector exerting activity both on its own and in combination with VX809 or GLPG/ABBV-2222.

## Introduction

Cystic fibrosis (CF) is a life-threatening, recessive genetic disease caused by mutations in a gene on chromosome 7 encoding for the CFTR protein. The CFTR protein is a cyclic adenosine monophosphate (cAMP)-regulated anion channel expressed primarily at the apical plasma membrane of secretory epithelia, and is responsible for the homeostasis of salt and water content on epithelial surfaces (Lubamba et al., 2012).

Cystic fibrosis transmembrane conductance regulator (CFTR) dysfunction causes the hallmark electrolyte elevation in sweat, and thick and viscous secretions affecting the lungs, reproductive tract and most exocrine glands, notably the pancreas, intestine, and bile duct. Individuals with CF present with multisystem disease involving several or all of these organs. However, pulmonary disease remains the leading cause of morbidity and mortality in CF, resulting from dehydration of the airway surface liquid and impaired airway mucociliary clearance, which leads to airway obstruction, chronic bacterial infection, chronic excessive inflammation, bronchiectasis and progressive decline in pulmonary function (O’Sullivan and Freedman, 2009; Lubamba et al., 2012; Okiyoneda et al., 2013; Pittman and Ferkol, 2015).

Nearly 2000 mutations in the CFTR gene have been identified [CF mutation database ^1^ and The Clinical and Functional TRanslation of CFTR (CFTR2) ^2^]. Overall, the broad spectrum of mutations can be grouped into six classes based on their effect on CFTR expression and/or function as shown in O’Sullivan and Freedman (2009), Boyle and De Boeck (2013), and Pittman and Ferkol (2015). CFTR mutations result in either no CFTR on the cell surface (Class I), almost no CFTR on the cell surface and with poor function (Class II), CFTR channels at the cell surface but failing to open in response to intracellular signals (no function, gating defect, Class III), CFTR channels at the cell surface that open but with reduced conductance (Class IV), or a reduced amount of CFTR on the cell surface (Class V and Class VI).

The majority of disease-causing mutations lead to misfolding of CFTR, including the deletion of phenylalanine at position 508, F508del, occurring in ∼90% of patient alleles (Bobadilla et al., 2002; Castellani et al., 2008). Misfolding results in a defective channel and the mutant protein either is released to the cell surface, where it will display functional defects, or is retained in the ER and retrotranslocated into the cytosol for degradation by the proteasome. Deletion of this phenylalanine (F508) in NBD1 causes not only NBD1 to misfold (Du et al., 2005), but also causes domain assembly defects (Protasevich et al., 2010; Thibodeau et al., 2010; Wang et al., 2010). As a result, the stability of the other domains is reduced, emphasizing the importance and sensitivity of CFTR’s interdomain contacts. It is not enough to preserve the polypeptide backbone at position F508 for proper folding: specific side-chain interactions at this site are needed as well for CFTR domain assembly (Thibodeau et al., 2005).

Classically, the mainstay treatments for CF patients were “downstream” symptomatic treatments to either improve airway clearance from thick secretions (e.g., hypertonic saline, dornase alpha), to treat acute and chronic pulmonary infections, to improve nutrition and digestion (e.g., high-calorie supplements, pancreatic enzymes), or to reduce inflammation (O’Sullivan and Freedman, 2009).

Recently, a limited set of disease modifying reagents, which are called CFTR modulators, became available. These CFTR modulators improve the processing and activity of the defective CFTR channels and are expected to lead to greater improvements in health and life-expectancy of CF patients (Hanrahan et al., 2013). Different types of small-molecule CFTR modulators are being developed: These include conformational stabilizers, commonly referred to as *corrector molecules* or “pharmaco-chaperones” that are designed to restore protein folding and allow protein maturation resulting in increased surface expression (Hanrahan et al., 2017) and *potentiator molecules* that increase the open probability of the channel (i.e., “gating function”) (Jih et al., 2017; Kym et al., 2018).

There are currently three approved CFTR modulator treatments available for CF patients, namely the potentiator Ivacaftor (Kalydeco^®^) or VX770, the corrector Lumacaftor or VX809 and the corrector Tezacaftor or VX661. The Ivacaftor/Lumacaftor combination therapy (Orkambi^®^) or the Ivacaftor/Tezacaftor combination therapy (Symdeko^®^) are available for the treatment of patients homozygous for the F508del CFTR mutation. However, clinical benefits from these treatments were somewhat limited (Wainwright et al., 2015; Taylor-Cousar et al., 2017). Thus there is a demand for improved combinations to further improve clinical benefit for CF patients with the F508del mutation.

It is well recognized that rescue of F508del CFTR to a clinically meaningful extent requires the combination of correctors and potentiators. In fact, more than one type of CFTR corrector may be required to significantly improve biogenesis of F508del CFTR. Some molecules are indeed described to further improve partially rescued F508del with VX809 or similar type 1 correctors. These molecules are shown to either indirectly improve the stability or trafficking of VX809 corrected F508del CFTR (Qian et al., 2015; Carlile et al., 2016; Giuliano et al., 2018) or directly improve F508del CFTR trafficking (Pedemonte et al., 2005; Van Goor et al., 2006; Pesci et al., 2015; Nieddu et al., 2016; Vu et al., 2017; Liu et al., 2018). Indeed, Vertex Pharmaceuticals has demonstrated the proof of concept that triple combination therapy regiment that adds a complementary-acting next-generation corrector to Symdeko formula results in significant clinical benefit in patients carrying the F508del mutation (VERTEX, 2017).

Here, we describe the identification and characterization of GLPG/ABBV-2737 (hereafter referred to as GLPG2737 or ‘2737), a corrector modulating folding and trafficking of F508del CFTR which exerts corrector activity on its own and additive to other correctors such as VX809, VX661, and GLPG/ABBV-2222 (herein referred to as GLPG2222 or ‘2222). GLPG2737 appears to have a novel mechanism of action, different to what has been described until now.

## Materials and Methods

### Materials

Following compounds were used for the generation of the different data. GLPG1837, GLPG3067, and GLPG2451 are potentiators improving the CFTR channel open probability. GLPG2222 is a type I corrector (similar to VX809 mechanism). All these compounds are/were in development by Galapagos and/or AbbVie.

### Cell Culture

A CFBE41o- cell line stably expressing F508del CFTR harboring an HRP-tag in the fourth extracellular loop was obtained from Professor Gergely Lukacs (Department of Physiology, McGill University, Montreal, QC, Canada) (Veit et al., 2012). Cells were grown in Eagle’s minimal essential medium (MEM) (Life Technologies) supplemented with 10% FBS, 1% L-glutamine (Life Technologies), 10 mM HEPES (Life Technologies), 200 μg/ml geneticin (Life Technologies) and 3 μg/ml puromycin (Sigma) in culture flasks coated with 0.01% bovine serum albumin (BSA) (Sigma), 30 μg/ml Purecol (Nutacon) and 0.001% human fibronectin (Sigma). HEK293 cells were cultured in uncoated flasks using Dulbecco’s Modified Eagle Medium (DMEM) (Life Technologies) supplemented with 10% FBS and 1% penicillin/streptomycin. The U2OS EA-MEM F508delCFTR cell line expresses the larger beta-galactosidase EFC fragment localized in the plasma membrane (EA: enzyme acceptor) and CFTR with the EA-MEM fusion protein, and was obtained from DiscoverX. These cells were cultured in a medium developed by DiscoverX (assay complete medium, 92-0018GK3). CHO cells were cultured in DMEM containing 10% FBS.

### Human Bronchial Epithelial (HBE) Cell Culture

Bronchial epithelial cells isolated from transplanted lungs from normal (wt CFTR) or CF patients homozygous for the F508del CFTR mutation, were obtained from McGill University (Montreal, QC, Canada) and University of North Carolina (Chapel Hill, NC, United States). Cells were isolated from lungs obtained from donors undergoing a planned transplantation. These primary cells were cultured directly on type IV collagen-coated polycarbonate Transwell supports with a diameter of 6.5 mm and pore size of 0.4 μm (Costar, #3397) for 18–25 days in air liquid (ali) interface essentially as previously described (Fulcher et al., 2005) for TECC. A similar cell culture protocol was used for Ussing chambers but using type IV collagen-coated polycarbonate Millicell supports with a diameter of 12 mm and a pore size of 0.4 μM (Millicell, PIHP01250).

### Western Blot (Band B/C)

For Western blot, U2OS EA-MEM F508del CFTR (CSE-MEM) cells were seeded in six well plates at a density of 2.5e6 cells/well on day 1 and incubated at 37°C and 5% CO_2_. Cells were treated with corrector compounds on day 2 and were lysed 24 h later. For the lysis, cells were washed with 1 ml PBS and incubated with 300 μl cell dissociation solution/well (Sigma, Cat No. C5789-100ML) for 15 min at 37°C and 5% CO_2_. Medium was collected and centrifuged for 5 min at 1,000 rpm and the supernatant removed. The pellet was re-suspended for 30 min on ice in the lysis buffer containing 1% NP-40, 0.5% sodium deoxycholate, 200 mM NaCl, 10 mM Tris pH 7.8, 1 mM EDTA and a protease inhibitor cocktail tablet from Roche (Cat No. 11873580001). For gel electrophoresis, 2.5 μl 10X Nupage reducing agent (Invitrogen, Cat No. NP0004) and 6 μl 4X Nupage sample buffer (Invitrogen, Cat No. NP0007) were added to 17 μl lysate and incubated for 15 min at 37°C. Twenty-five microliters of sample was loaded on a 7% Tris acetate gel (Invitrogen, Cat No. EA0355BOX) using Tris acetate SDS running buffer (Invitrogen, Cat No. LA0041) during a period of 80 min at 150 V. Transfer was performed using a PVDF membrane and a transfer buffer from Nupage (Invitrogen, Cat No. NP0006-1) for 90 min at 90 V. Afterward the PVDF membrane was washed using 0.1% (v/v) Tween20 in PBS (PBST) for 15 min and blocked using 5% (w/v) skimmed milk in PBST for 2 h. The membrane was incubated overnight with 5 ml primary antibodies (596 anti-CFTR from the CF foundation at 1/500 dilution and Anti-alpha 1 Na/K-ATPase (Abcam ab7671) at 1/2000 dilution) in milk-PBST at 4°C. The next day, membranes were washed three times for 5 min with PBST and incubated with a secondary antibody (polyclonal goat anti-mouse immunoglobulins/HRP, Dako P0447) for 1 h at 4°C. After washing three times for 5 min with PBST, the blot was developed by incubating with Super Signal West Dura Extended duration substrate (2.5 ml peroxide buffer + 2.5 ml luminol/enhancer, Thermo Scientific, Cat No. 34075) for 5 min at room temperature. A picture was taken using the chemiDoc touch imaging system from BIO-RAD at an automatic exposure setting.

### Yellow Fluorescent Protein (YFP) Halide Assay

HEK293 cells were transfected with 10–80 ng of plasmid encoding F508del, E92K, P67L, V232D, F508del/G550E, F508del/I539T or wild type CFTR and 20 ng of plasmid encoding YFP (H148Q/I152L/F47L) using jetPEI (Polyplus transfection). Directly after transfection, cells were seeded in black 96-well plates coated with poly-D-lysine at a density of 70,000 cells per well. The next day, cells were treated with corrector(s) for 24 h. After this incubation period, cells were washed twice with DPBS with Ca^2+^ and Mg^2+^. Subsequently, cells were treated with 10 μM forskolin and the desired concentration of potentiator in a volume of 40 μl and incubated at room temperature for 10 min, a time point optimized in previous experiments resulting in a good window (positive control/negative control > 2) and signal to background ratio. YFP fluorescence was measured using an EnVision plate reader (PerkinElmer). The signal was recorded for 7 s, starting just before injection of 110 μl NaI buffer (137 mM NaI, 2.7 mM KI, 1.7 mM KH_2_PO_4_, 10.1 mM Na_2_HPO_4_, 5 mM D-glucose) into the well with a speed of 150 μl/s, resulting in a final volume of 150 μl. The excitation wavelength was 485 nm and the emission wavelength 530 nm. The capacity of potentiators to increase CFTR channel function was expressed as 1-(fluorescence 7 s after NaI addition (F)/fluorescence before NaI addition (F0)). For the different mutants, data was normalized using the formula: normalized response = 100^∗^(absolute response – negative control response)/(GLPG2222 response – negative response) as such, GLPG2222 response corresponds to 100. Concentration–response experiments were fitted using a 4 parameter hill function of the form Response = Bottom + (Top - Bottom)/(1 + (EC_50_/concentration) ˆHillSlope)) to determine EC_50_ values.

### Cell Surface Expression Horseradish Peroxidase Assay (CSE-HRP Assay)

CFBE41o^-^ TetON cells expressing HRP tagged F508del-CFTR (Veit et al., 2012) were seeded at a density of 2000 cells per well in white 384-well plates (Greiner). Medium containing 500 ng/ml doxycycline was used to induce expression of the F508del-CFTR–HRP construct. After 3 days, cells were treated with corrector and/or potentiator compounds and transferred to an incubator at 33°C. On day 4, cells were washed five times with PBS containing Ca^2+^ and Mg^2+^ using a Bio-Tek plate washer and incubated with a chemiluminescent HRP substrate (SuperSignal West Pico Chemiluminescent Substrate, Thermo Scientific) for 15 min. Chemiluminescence was measured using an Envision plate reader (Perkin Elmer).

### Cell Surface Expression DiscoverX Assay (CSE-MEM Assay)

Twenty-five microliters of cell plating reagent 5 (DiscoverX, 93-0563R5A) containing 5000 U2OS EA-MEM F508del_CFTR cells per well was added in white 384 well plates (Greiner, 781080) on day 1 and incubated overnight at 37°C, 5% CO_2_. On day 2, medium was refreshed and compounds (1 μl of 26 × concentrated solution) were added to the cells. All compounds used during the experiment were first dissolved in DMSO to a 10 mM solution which was then diluted to a 26 × solution in the medium just prior to the experiment. Cells were incubated with compounds for 20–24 h at 37°C, 5% CO_2_. On day 3, the plates were first incubated at room temperature for 1 h. After 1 h the plates were washed two times with PBS +/+ (Gibco, 14040-091) and 30 μL PBS +/+ (Gibco, 14040-091) was added after the last wash step. Then 15 μL of substrate (DiscoverX, 93-0247: 1 part Flash cell assay buffer + 4 parts Flash substrate) was added per well. After 1 h of incubation at room temperature in the dark, the luminescence signal was read on an EnVision plate reader (PerkinElmer).

### TECC Experiments

Corrector and/or potentiator compound(s) were added on the basolateral side of the HBEs for 24 h to rescue F508del CFTR. *Trans*-epithelial clamp circuit (TECC) recordings were performed using the TECC instrument developed and sold by EP Design (Bertem, Belgium). During the recording, the epithelial cells were bathed in a NaCl-Ringer solution (120 mM NaCl, 20 mM HEPES, 1.2 mM CaCl_2_, 1.2 mM MgCl_2_, 0.8 mM KH_2_PO_4_, 0.8 mM K_2_HPO_4_, 5 mM glucose, pH 7.4) on both the basolateral (640 μl) and the apical side (160 μl) and kept at 37°C. Apical amiloride was used to inhibit the endogenous ENaC currents (100 μM) while forskolin (10 μM) was applied on both the apical and basolateral sides to stimulate CFTR. All triggers and compounds used during the experiment were first dissolved in DMSO to a 1000 × concentrated solution, just prior to treatment a 10 × stock was prepared in the NaCl-Ringer solution which was used for addition of the correct concentration of trigger and/or compound during the experiment. Measurements were done during a 20-min timeframe with recordings every 2 min. The transepithelial potential (PD) and transepithelial resistance (*R_t_*) were measured in an open circuit and transformed to *I_eq_* using Ohm’s law. The maximal increase in *I_eq_* (Δ *I_eq_*, the difference in current before and after FSK and/or potentiator treatment) was used as a measure for the increased CFTR activity. EC_50_ values were generated by measuring impact of different concentrations of compound on *I_eq_* in primary cells. For this purpose each transwell was treated with a different compound concentration. CFTRInh-172 was added apically at 10 μM to assess the specificity of the response. Dose response data was fitted using a 3 parameter hill function of the form Response = Bottom + (Top - Bottom)/(1 + (EC_50_/concentration)) to determine EC_50_ values.

### Patch-Clamp Electrophysiological Recording

A more complete description of the patch-clamp methodology can be found in a recent publication (Lin et al., 2016). Briefly, CHO (Chinese Hamster Ovary) cells were transiently transfected with pcDNA plasmids containing a F508del CFTR construct and pEGFP-C3. After transfection, cells were trypsinized and plated onto 35 mm tissue culture dishes dispersed with sterilized glass chips. Electrophysiological experiments were performed 3–7 days following transfection.

Patch-clamp experiments were performed at room temperature with an EPC-9 patch clamp amplifier (HEKA Instruments, Holliston, MA, United States). Recording microelectrodes were made from borosilicate capillary glass with a two-stage pipette puller (Narishige, Tokyo, Japan). The pipette tip was polished with a home-made microforge before use. The pipette solution contained (in mM) 140 NMDG (*N*-methyl-D-glucamine)-Cl (Fisher Biotec), 2 MgCl_2_ (Fisher Biotec, Perth, WA, Australia), 5 CaCl_2_ (Fisher Biotec), and 10 HEPES (Fisher Biotec), pH 7.4, with NMDG (see Lin et al., 2016 for details). The pipette resistance when filled with the regular pipette solution was 3–5 MΩ. Once the tip of the pipette and the cell membrane established GΩ resistance, the electrode was quickly pulled away from the cell to form an inside-out patch, where the cytosolic side of the membrane was exposed to the perfusion solution. Cells were perfused with a bath solution having (in mM) 145 NaCl (Fisher Biotec), 5 KCl (Fisher Biotec), 2 MgCl_2_, 1 CaCl_2_, 5 glucose (Fisher Biotec), 5 HEPES, and 20 sucrose (Fisher Biotec), pH 7.4, with NaOH (Fisher Biotec). After establishing an inside-out configuration, the patch was perfused with a standard perfusion solution (i.e., intracellular solution) containing (in mM) 150 NMDG-Cl, 2 MgCl_2_, 10 EGTA (Fisher Biotec), and 8 Tris (Fisher Biotec), pH 7.4, with NMDG (see Lin et al., 2016 for details) containing the catalytic subunit of protein kinase A (PKA) and 2 mM ATP. Once the phosphorylation-dependent activation of CFTR reached a plateau, 10 μM GLPG3067 was applied in the continuous presence of 2 mM ATP until a steady state was attained.

Microscopic kinetic analysis was performed with a program provided by Dr. László Csanády (Semmelweis University, Budapest, Hungary) (Csanády, 2000). Experiments were performed four to five times and averages and SEM were calculated. The resulting Open probability (Po) values and single-channel kinetic parameters were compared with paired *t*-test (Excel, Microsoft); *P* < 0.05 is considered statistically significant.

### Ussing Chamber Experiments

Corrector compound(s) and potentiator were added in the culture medium on the basolateral side of the F508del/F508del HBE cells for 24 h to partially rescue F508del CFTR. Ussing chamber experiments were performed using chambers developed by physiological instruments. During the recording, the epithelial cells were kept at 37°C and bathed on the basolateral side in 4 ml of a NaCl-Ringer solution (120 mM NaCl, 25 mM NaHCO_3_, 1.2 mM CaCl_2_, 1.2 mM MgCl_2_, 0.8 mM KH_2_PO_4_, 0.8 mM K_2_HPO_4_, 5 mM glucose, pH 7.4) and 4 ml of a low chloride solution on the apical side (120 mM Na-glutamate, 25 mM NaHCO_3_, 1.2 mM CaCl_2_, 1.2 mM MgCl_2_, 0.8 mM KH_2_PO_4_, 0.8 mM K_2_HPO_4_, 5 mM glucose, pH 7.4). Apical amiloride was used to inhibit the endogenous ENaC currents (100 μM) while forskolin (10 μM) was applied on both the apical and basolateral sides to stimulate CFTR. All triggers and compounds used during the experiment were first dissolved in DMSO to a 1000 × concentrated solution, just prior to treatment. Measurements of the FSK response were done during a 20-min timeframe with recordings every second. The short circuit current (*I_sc_*) was measured and the increase in *I_eq_* (Δ *I_eq_*, the difference in current before and after FSK and potentiator treatment) was used as a measure for the increased CFTR activity. CFTRInh-172 was used at 10 μM to assess the specificity of the response.

## Results

### Discovery of a Novel Corrector Series

To discover novel correctors several high throughput screening campaigns were performed using cell based assays measuring plasma membrane expression of the F508del CFTR protein. The CSE-HRP system used CFBE41O-cells, a lung epithelial cell line, stably transfected with F508del CFTR tagged with HRP in the fourth extracellular loop (Veit et al., 2014). The CSE-MEM system, used U2OS cells, an osteosarcoma epithelial cell line, stably transfected with F508del CFTR tagged with a Pro-link moiety at the C-terminal and a plasma membrane located EA (enzyme acceptor) moiety. The latter cells were created by DiscoverX and the assay further optimized to allow robust evaluation in a high throughput manner.

For screening, cells were pre-incubated with compound for 24 h prior to read-out to allow sufficient time for *de novo* synthesis, and cellular trafficking of F508del-CFTR to reach steady state levels at the plasma membrane. A diverse set of 105,000 small molecules was screened in both cell assays in presence and absence of a fixed concentration (3 μM) of VX809. For the identification of suitable starting points we primarily focused on hits that increased the F508del CFTR surface expression under all conditions as exemplified by compound HIT1.

**FIGURE 1:**
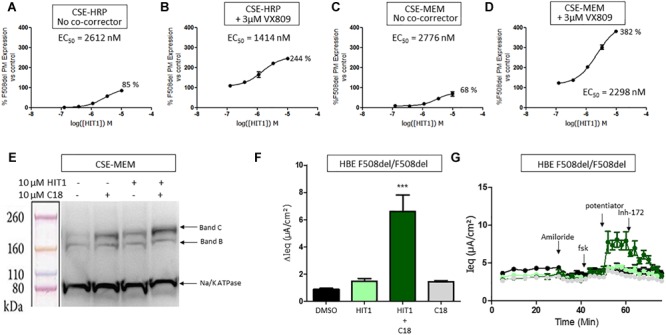
Effect of HIT1 on F508del CFTR trafficking and function. **(A)** Dose response of 24 h treatment with HIT1 in CSE-HRP assay, % F508del CFTR expression at plasma membrane was normalized using VX809 correction as 100% in **(A–D)** and each concentration was tested in duplicate. **(B)** Dose–response of 24 h treatment with HIT1 in combination with 3 μM VX809 in CSE-HRP assay. **(C)** Dose–response of 24 h treatment with HIT1 in CSE-MEM assay. **(D)** Dose–response of 24 h treatment with HIT1 in combination with 3 μM VX809 in CSE-MEM assay. **(E)** CSE-MEM cells were incubated with compound for 24 h. Band C, mature complex-glycosylated F508del CFTR; Band B, immature core-glycosylated CFTR. **(F)** F508del/F508del HBE cells were incubated with compound for 24 h, CFTR channel was activated with 10 μM forskolin and 3 μM potentiator. ^∗∗∗^ denotes a *p*-value < 0.001 compared to DMSO treatment. **(G)** Average of raw traces of experiments represented in F (*n* = 4 for each condition in **F** and **G**).

HIT1 was able to rescue trafficking of F508del CFTR to the plasma membrane in both the CSE-HRP and CSE-MEM assay reaching efficacies of 85% and 68%, respectively, compared to VX809 rescue (100%) with micromolar potency (Figure 1A,C). When combined with 3 μM VX809, the rescued F508del CFTR at the plasma membrane increased approximately to 244% (CSE-HRP) and 382% (CSE-MEM) of that of VX809 while retaining similar potency (Figure 1B,D).

To confirm that HIT1 affects F508del CFTR protein folding and trafficking, a western blot was performed to examine the expression and maturation of F508del CFTR after correction. As illustrated in Figure 1E, treatment of CSE-MEM cells with HIT1 resulted in a clear increase of the more complex glycosylated form of CFTR, i.e., band C, supporting that HIT1 is able to partially rescue the F508del folding defect. An additive increase in Band C was obtained when combining HIT1 with C18, a well characterized VX809 analog provided by Cystic Fibrosis Foundation Therapeutics Inc. (CFFT).

In a next step, we wanted to determine if HIT1 also restored the chloride transporter activity of the rescued F508del CFTR in primary human broncho-epithelial cells (HBE’s). For this, HBE cells derived from an F508del/F508del CF patient grown in ALI for 21 days were treated for 24 h with HIT1 alone or in combination with C18 corrector. As shown in Figure 1F,G, treatment with 10 μM HIT1 resulted in only a minor increase of the *I_eq_*. However, in combination with 10 μM C18, a significant increase in *I_eq_* compared to DMSO treated control cells or cells treated with C18 alone, was measured after stimulation with 10 μM forskolin (FSK) and 3 μM of an in house potentiator GLPG1837 (Van der Plas et al., 2018) (*n* = 4 for each treatment condition). These concentrations were selected based on indications from previous experiments (Yeh et al., 2017; Gees et al., 2018).

**FIGURE 2:**
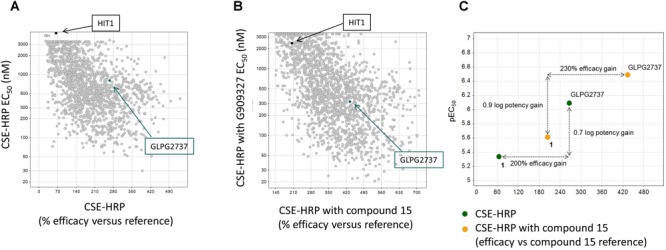
Progression of efficacy (*X*-axis) and potency (EC_50_, *Y*-axis) of corrector series on F508del CFTR, in a CSE-HRP assay. Each dot represents a single compound. Control used in assay corresponds to compound 15 (a lead compound derived from GLPG2222 series, Wang et al., 2018). **(A)** Effect of compounds on their own on F508del CFTR rescue. **(B)** Effect of compounds when combined with a C1 corrector (compound 15). Black dot represents the original hit HIT1, the green dot represents GLPG2737. **(C)** Schematic representation of the shifts in potency and efficacy comparing Hit1 with GLPG2737 both with and without co-corrector treatment.

Collectively the above data show that HIT1 treatment, alone or in combination with Type I corrector, i.e., C18, results in a functional rescue of the F508del folding defects, making it an attractive starting point for drug discovery. Several rounds of medicinal chemistry optimization were used to improve the *in vitro* potency as well as efficacy and build up a structure activity relationship (SAR) around the HIT1 scaffold (Figure 2). Throughout this process, drug-like and pharmacokinetic properties were also assessed and optimized which led to the selection of GLPG2737 as the molecule having the right balance between drug-like properties and biological activity.

### GLPG2737 Pharmacology

**FIGURE 3:**
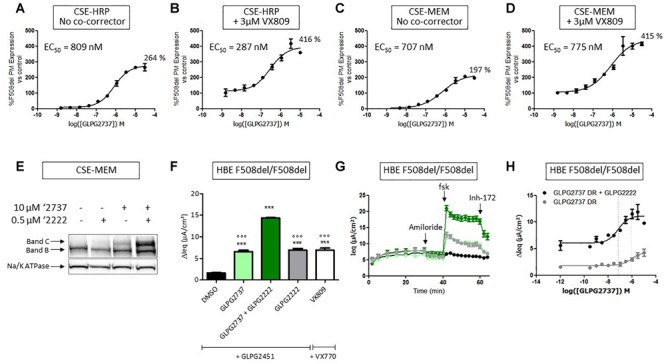
Effects of GLPG2737 on F508del CFTR maturation and function. **(A)** Dose–response of GLPG2737 after 24 h treatment in CSE-HRP assay, % F508del CFTR expression at plasma membrane (PM) was normalized to control compound 15 (derived from GLPG2222 series) in **(A–D)** and each concentration was tested in duplicate. **(B)** Dose–response of GLPG2737 in combination with 0.5 μM compound 15 (derived from GLPG2222 series) after 24 h treatment in CSE-HRP assay. **(C)** Dose–response of GLPG2737 after 24 h treatment in CSE-MEM assay. **(D)** Dose–response of GLPG2737 in combination with 0.5 μM GLPG2222 after 24 h treatment in CSE-MEM assay. **(E)** CSE-MEM cells were incubated with compound for 24 h. Cell lysates were loaded on gel and Band B/C was detected using 596 antibody (Band C, mature complex-glycosylated F508del CFTR; Band B, immature core-glycosylated CFTR. **(F)** F508del/F508del HBE cells were incubated with indicated corrector compound(s) for 24 h, CFTR channel was activated with 10 μM forskolin and 1.5 μM potentiator. ^∗∗∗^ denotes a *p*-value < 0.001 compared to DMSO treatment. ^∘∘∘^ denotes a *p*-value < 0.001 compared to GLPG2222 + GLPG2737 treatment. **(G)** Average of raw traces of experiments represented in F (*n* = 3–6 for each condition in **F** and **G**) **(H)** Dose–response of 24 h treatment with GLPG2737 in combination with 0.15 μM GLPG2222 (black) or without (gray) and 1.5 μM GLPG3067 (tested in duplicate for each concentration).

When used as a single agent, GLPG2737 is a nanomolar potent pharmaco-chaperone of F508del CFTR in different assays, reaching efficacies of 264% (CSE-HRP) and 197% (CSE-MEM), compared to VX809 rescue (100%) (Figure 3A,C). When combined with 3 μM VX809, the amount of rescued F508del CFTR further increased to 416% (CSE-HRP) and 415% (CSE-MEM) relative to VX809 (Figure 3B,D). In addition to its effect in the primary assays, the GLPG2737 corrector activity was also confirmed in Band B/C assays (Figure 3E) and functional consequences of that was further investigated in HBE’s using TECC. As it is well recognized that rescue of F508del CFTR to a clinically meaningful extent requires the combination of various correcting approaches, we evaluated GLPG2737 on top of the corrector GLPG2222 (Wang et al., 2018). As shown in Figure 3H, addition of GLPG2737, significantly increased the Cl currents to a level of 182 ± 13% of the GLPG2222 treated cells with a potency of 18 ± 6 nM (average of eight repeats in four different donors, representative curve in Figure 3H, donor specific data in Supplementary Table 1). Similar responses were obtained using the ‘gold’ standard Ussing chamber in asymmetric buffers on HBE cells (Supplementary Figure 1). Figure 3F shows Cl currents obtained with dual combinations of GLPG2222 with GLPG2451, GLPG2737 with GLPG2451 and VX809 with VX770 as well as the further increase in Cl current when using the triple combination of GLPG2222, GLPG2737, and GLPG2451.

**FIGURE 4:**
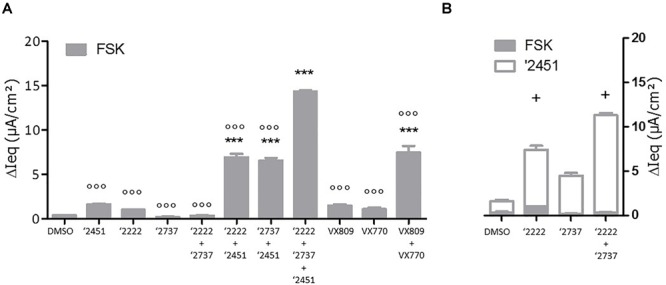
Effects of single corrector or combinations of correctors on F508del CFTR channel function as measured by TECC in F508del/F508del HBE cells. **(A)** Compounds (0.15 μM GLPG2222 and/or 1 μM GLPG2737 and/or 1.5 μM GLPG2451) were incubated on cells for 24 h, CFTR channel was activated with 10 μM forskolin and change in short circuit current was measured in three to six replicates for each condition ^∗∗∗^ denotes a *p*-value < 0.001 compared to DMSO treatment. ^∘∘∘^ denotes a *p*-value < 0.001 compared to GLPG2222 + GLPG2737 + GLPG2451 treatment. **(B)** Compounds (0.15 μM GLPG2222 and/or 1 μM GLPG2737) were incubated on cells for 24 h, CFTR channel was activated with 10 μM forskolin followed by 1.5 μM potentiator GLPG2451 and change in short circuit current was measured after forskolin activation and after GLPG2451 addition. *n* = 3 for each condition and *p*-values are denoted with + meaning *p* < 0.01 comparing similar chronic conditions from panel **(A)** with acute potentiator conditions in panel **(B)**.

To gain further insight into the contribution of each component, cells were pre-treated with different corrector combinations for 24 h and TECC measurements were performed upon sequentially addition of FSK and the potentiator GLPG2451 (Figure 4). After FSK stimulation, GLPG2222 showed a small but reproducible signal which was not observed for GLPG2737 (Figure 4A). In contrast, GLPG2737 slightly decreased the FSK induced response compared to DMSO control. Interestingly the FSK effect of GLPG2222 was lost when combined with GLPG2737. However, after potentiator treatment, both GLPG2737 and GLPG2737 + GLPG2222 treated cells showed a significant increase in current (Figure 4).

Next we looked at the effect of GLPG2222 on the potency of GLPG2737. While the EC_50_ of GLPG2737 in F508del/F508del HBE cells was 497 ± 189 nM (*n* = 6 in three different donors homozygous for the F508del CFTR mutation) when combined with a potentiator, the GLPG2737 potency when combined with GLPG2222 (0.15 μM) and a potentiator shifted approximately ∼25 fold to 18 ± 6 nM (*n* = 8 in four different donors homozygous for the F508del CFTR mutation) (Figure 3H). These data provided further support for both correctors to be interlinked. As a note, GLPG2451 and GLPG3067 are potentiators derived from the same chemical series exerting similar biological activity, data generated with either can be compared.

### Effect of GLPG2737 on Open Probability of F508del CFTR

**FIGURE 5:**
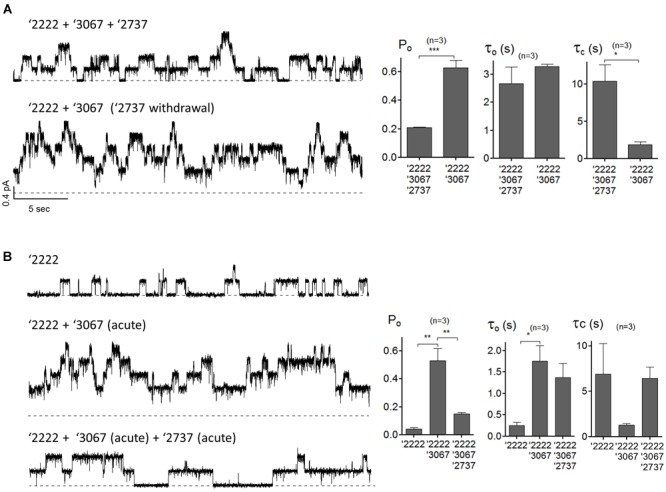
Single-channel behaviors of F508del CFTR expressed in CHO cells pre-treated with corrector(s) GLPG2222, GLPG2737 and or potentiator GLPG3067. **(A)** Representative single-channel current traces of F508del CFTR in the presence of 0.5 μM GLPG2222 + 10 μM GLPG3067 + 1 μM GLPG2737 (upper trace) and after washout of GLPG2737 (lower trace) from a cell pre-treated with 0.5 μM GLPG2222 + 10 μM GLPG3067 + 1μM GLPG2737. Kinetic parameters (Po, τ_o_, and τ_c_) are shown on the right. **(B)** Representative single-channel current traces of F508del CFTR in the presence of 0.5 μM GLPG2222 (upper panel), after addition of 10 μM GLPG3067 (middle panel) and additional application of 1 μM GLPG2737 (lower panel) from a cell pre-treated with 0.5 μM GLPG2222. Kinetic parameters (Po, τ_o_, and τ_c_) are shown on the right. The statistical analysis of single-channel kinetic parameters was described in “Materials and Methods” Section, every condition was tested at least three times. ^∗^*P* < 0.05; ^∗∗^*P* < 0.01; ^∗∗∗^*P* < 0.005.

The observation that GLPG2737 alone or in combination with GLPG2222 did not result in a forskolin induced current was surprising to us and therefore further evaluations were done in order to better understand what caused this effect. To this end, patch clamp experiments were performed in cells over-expressing F508del CFTR pretreated with correctors with or without addition of a potentiator GLPG3067. Presumably because of a large increase of the number of F508del CFTR channels at the plasma membrane, microscopic recordings are hard to get. Nonetheless, Figure 5A shows a representative current trace from a patch containing few channels from cells pre-treated with corrector GLPG2222 (0.5 μM), corrector GLPG2737 (1 μM) and potentiator GLPG3067 (10 μM) for 24 h, and these correctors were added in the recording solution. Kinetic analysis shows an apparent Po of 0.21 ± 0.003 with τ_o_ = 2.68 ± 0.59 s and τ_c_ = 10.35 ± 2.22 s (Figure 5A). While this Po was a clear increase compared to untreated control cells (Po = 0.04, Gees et al., 2018), interestingly the channel activity was increased to 0.63 ± 0.05 after washout of GLPG2737. Of note, this change of Po is mainly due to a shortening of the closed time τ_c_ (10.35 ± 2.22 s with GLPG2737 vs. 1.89 ± 0.38 s without) with a minimal change in the open time τ_o_ (2.68 ± 0.59 s with GLPG2737 vs. 3.28 ± 0.09 s without). These data suggest a possible inhibitory action of GLPG2737 on CFTR gating. We therefore tested the effect of acute GLPG2737 addition in inside-out patches (Figure 5B). When cells were treated for 24 h with GLPG2222 (0.5 μM) there was only a minor increase in Po to 0.04 ± 0.01 with τ_o_ = 0.25 ± 0.08 s and τ_c_ = 6.88 ± 3.36 s (n = 3). Only after the direct addition of the potentiator GLPG3067 (10 μM) there was a clear increase in Po to 0.53 ± 0.09 in these patches (τ_o_ = 1.76 ± 0.36 s and τ_c_ = 1.27 ± 0.16 s). Addition of GLPG2737 (1 μM) to these patches resulted in a decrease of the Po to 0.15 ± 0.01. Again, this was accompanied by a change in τ_c_ (6.42 ± 1.24 s with GLPG2737 vs. 1.27 ± 0.16 s without) with only a minor change in τ_o_ (1.37 ± 0.33 s with GLPG2737 vs. 1.76 ± 0.36 s without). Together, these data suggest that GLPG2737, while an effective corrector, can act as a gating inhibitor of F508del CFTR mainly by increasing the τ_c_. A similar observation was made on WT CFTR with a different compound from the same series (data not shown).

### GLPG2737 Inhibits Wild Type CFTR Channel Activity

**FIGURE 6:**
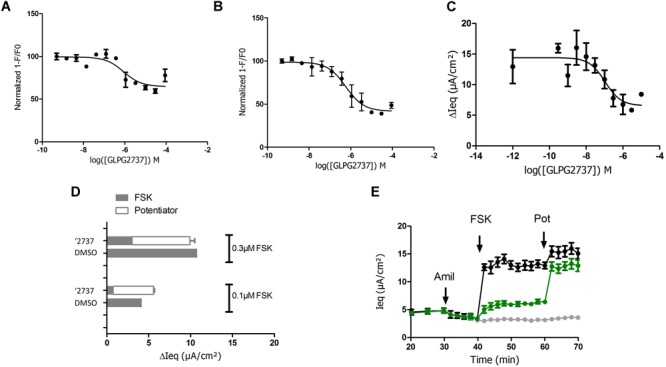
Effects of GLPG2737 on normal human bronchial epithelial cells (NHBE) and HEK cells expressing WT CFTR. GLPG2737 dose response effect on WT CFTR activity in HEK293 cells after 24 h stimulation **(A)** or acute incubation **(B)** and activation using 100 nM FSK. **C.** GLPG2737 dose response effect on CFTR activity in NHBE cells after acute addition (0.3 μM forskolin) (tested in duplicate at each concentration for panels **A–C**). **(D)** NHBE treated for 24 h with 1 μM GLPG2737 or DMSO (control), CFTR channel was activated with either 0.1 μM or 0.3μM forskolin, measured *I_sc_* shown in gray. After forskolin treatment, potentiator GLPG2451 (10 μM) was added to the GLPG2737 treated cells, further increase in *I_sc_* shown in white bar. *n* = 2–3 for each condition, *p* < 0.01 when comparing GLPG2737 with DMSO treated cells after forskolin treatment (all cases) and *p* > 0.05 when comparing GLPG2737 with DMSO treated cells after forskolin + potentiator treatment (all cases). **(E)** Representation of the average raw traces represented in panel A (0.3 μM forskolin). Light gray line represents cells without forskolin. Green line represents GLPG2737 treated cells. Black line represents cells treated only with forskolin.

Due to the lack of any forskolin activated current upon correction of F508del carrying cells with GLPG2737, we wondered whether GLPG2737 would have an effect on wild type CFTR activity. To evaluate this, we developed a YFP halide assay using HEK293 cells transfected with wild type human CFTR (Galietta et al., 2001). Incubation of cells during 24 h with a dose range of GLPG2737 resulted in a dose dependent decrease in wild type CFTR activity (Figure 6A), suggesting GLPG2737 to be an inhibitor of CFTR while it also is a clear corrector of F508del CFTR. To support this hypothesis further, GLPG2737 was acutely added together with forskolin to wild type CFTR expressing cells. Similar to the observations in the 24 h incubation experiment, a decrease in wild type channel activity was measured with increasing amounts of GLPG2737 (Figure 6B). Since the YFP halide assay was performed using an overexpressing cell system that might not be physiologically relevant, GLPG2737 was also evaluated on normal HBE cells. Here as well, addition of GLPG2737 resulted in reduced CFTR activity which could be rescued upon addition of a potentiator (Figure 6C–E).

### GLPG2737 Can Rescue Other Than F508del CFTR Folding Mutants

**FIGURE 7:**
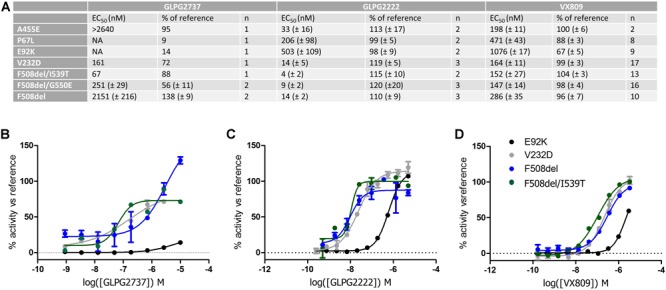
YHA assay using CFTR mutant overexpression in HEK293 cells. Cells were incubated for 24 h with either a dose range of GLPG2737, GLPG2222 or VX809. After washing, cells were triggered with forskolin (50 μM for F508del, E92K, V232D and F508del/I539T, 10 μM for P67L or 1 μM for F508del/G550E) and 0.5 μM potentiator and YFP fluorescence was measured. Efficacy was determined using 10 μM GLPG2222 as positive control and DMSO as negative control in the assay. **(A)** Table with potency and efficacy obtained for the mutants and compounds tested. **(B)** Dose–response curves of GLPG2737. **(C)** Dose–response curves of GLPG2222. **(D)** Dose–response curves of VX-809. For panels **(B–D)**, E92K mutant is represented in black, F508del mutant in blue, F508del/I539T mutant in green and V232D mutant in gray, all concentrations are tested in duplicate.

We then wondered whether GLPG2737 could rescue other CFTR folding mutants beyond F508del. For this, various YFP halide assays were developed overexpressing either F508del, E92K, P67L or V232D CFTR (Figure 7, Supplementary Figure 2). For further understanding and interpretation, we also tested GLPG2222 and VX809 in the same assays. GLPG2737 was able to rescue V232D CFTR with an EC_50_ of 161 nM (EC_50_ on F508del CFTR was 2.2 ± 0.2 μM, see above). Most striking was the absence of rescue of E92K and P67L by GLPG2737, while both mutants could be rescued by GLPG2222 and VX809 albeit with a reduced potency compared to F508del CFTR.

When testing the compounds on suppressor mutants F508del/G550E, F508del/I539T, a clear rescue was observed for all compounds albeit with GLPG2737 having a >10× improved EC_50_ compared to F508del CFTR while potency for GLPG2222 and VX809 was in the similar range on F508del CFTR. Both mechanisms of correction appear to act complementary to the suppressor mutations which partially rescue NBD1 misfolding suggesting both mechanisms not to interfere with the further improvement of the suppressor mutations. Both types of corrector seem to be able to rescue V232D in this assay.

## Discussion

There is a high unmet need for novel treatment options for patients with CF. As such, there is a clear interest to identify and develop additional CFTR modulators for the treatment of CF. It is generally well accepted that a combination of at least three molecules will be required to achieve significant clinical benefit (Clancy et al., 2012; Okiyoneda et al., 2013). This concept has recently been strengthened by the clinical data generated by Vertex pharmaceuticals where the addition of a next generation corrector (such as VX440, VX152, VX659 or VX445) to patients on stable treatment with Symdeko^®^led to an improvement in ppFEV1 of ≥10 percent points (VERTEX, 2017).

Nevertheless, the identification of potent, safe CFTR modulators has proven to be difficult and only few made it to the clinic (Pedemonte et al., 2005; Van Goor et al., 2006; Pesci et al., 2015; Qian et al., 2015; Carlile et al., 2016; Nieddu et al., 2016; Mijnders et al., 2017; Vu et al., 2017; Giuliano et al., 2018; Liu et al., 2018). Although targeting more indirect mechanism is possible, e.g., PTI-428 (PTI Therapeutics, 2017; Giuliano et al., 2018), interfering with F508del CFTR folding, seems currently to be the most promising concept to move forward, as it is only dependent on CFTR protein availability and to a lesser extent on the surrounding cellular context.

GLPG2737 has been discovered using novel screening technologies directly measuring the level of surface expression of F508del CFTR. In primary bronchial epithelial cells, GLPG2737 (when combined with a potentiator) shows a similar level of rescue as treatment with GLPG2222 + GLPG3067 and a further increase to 182% as part of the triple combination together with GLPG2222.

Interestingly the potency of the compound is strongly influenced by the presence of the co-corrector as the GLPG2737 in combination with GLPG2222 is about 25-fold more potent compared to GLPG2737 its own. This observation suggests GLPG2222 to bind first to F508del CFTR, partially rescuing it and bringing it in a conformation which allows GLPG2737 to better exert its biological action, however, further in depth studies would be required to demonstrate this.

Looking at the mechanism of action we noticed that the activity of GLPG2737 is strongly dependent on the presence of a potentiator. When evaluated in HBE cells using TECC as readout, we noticed that after forskolin addition (no potentiator present), no channel activity could be measured with GLPG2737, while GLPG2222 treatment resulted in a small but measurable signal and similar observations were made when GLPG2737 was in combo GLPG2222.

In contrast, combining GLPG2737 alone or together with GLPG2222 for 24 h in presence of a potentiator resulted in the expected level of F508del channel activity (based on observed F508del CFTR protein levels) showing functional rescue when present in dual or triple combinations with a potentiator.

To better understand this phenomenon, GLPG2737 was evaluated for its effect on wild type CFTR. Surprisingly wild type CFTR channel activity was inhibited in a dose dependent manner by GLPG2737, not only in a cellular assay with overexpression of CFTR, but also in human bronchial epithelial cells from non-CF donors. Similar to the effect observed on F508del CFTR, this inhibitory effect could be counteracted by the addition of a potentiator, suggesting GLPG2737 to directly influence the channel’s open probability. Indeed, patch clamp data (Figure 5) demonstrated that the triple combination (GLPG2737 + GLPG2222 + GLPG3067) shows a lower open probability of rescued F508del CFTR compared to the dual combination (GLPG2222 + GLPG3067).

Overall, the data obtained so far support a model whereby the addition of GLPG2737 to the corrector/potentiator combination GLPG2222/GLPG3067 greatly increases the number of CFTR F508del channels rescued to the plasma membrane, though with each channel having a somewhat reduced activity. It appears that GLPG2737 albeit being a very good corrector for F508del CFTR; it may exert its biological activity by bringing the channel into a more rigid conformation to the plasma membrane akin to G551D CFTR. Likely, by rigidifying the channel structure, it could bypass better the cell’s controlling mechanism allowing trafficking toward the plasma membrane. Like G551D CFTR, the F508del CFTR rescued by GLPG2737 requires the presence of a potentiator to become functional.

Taken together, treatment of patient derived primary bronchial epithelial cells with the triple combination (GLPG2737 + GLPG2222 + GLPG3067) leads to a substantial net increase in chloride transport compared to the dual combination (GLPG2222 + GLPG3067, Figure 3F,G, 4) which in the end is the parameter believed to translate into benefit for the patients.

GLPG2737 corrects F508del CFTR with a complementary mechanism to GLPG2222 or VX809, but also to NBD1 stabilizing mutants. The latter can also be further rescued by VX809 suggesting no interference of mechanisms of VX809 and GLPG2737 with correction of NBD1 suppressor mutants. One could speculate that if a triple combination of two correctors and a potentiator still not yields sufficient clinical benefit for the patients, a quadruple combination with the addition of a NBD1 stabilizing drug could further rescue F508del CFTR, hence potentially improve clinical benefit. The data using different CFTR mutants show differences in rescue depending on type of mutations, however, further studies are required to understand better how GLPG2737 is able to rescue F508del (and other mutant) CFTR at a molecular level. This work represented at high level the potential differences in mechanism of action between correctors, but making speculations on the potential binding site or mechanism of action is not feasible based on current data.

Altogether, GLPG2737 represents a novel class of CFTR correctors with attractive biological features supporting its further progression toward the clinic. GLPG2737 has gone through an extensive safety profiling and was subsequently dosed to healthy volunteers. The observed exposure of GLPG2737 in humans was dose linear and allows for a once daily dosing regimen in patients (Brearley et al., 2017). Currently, GLPG2737 is being evaluated in two patient studies (van Koningsbruggen-Rietschel et al., 2018). One study focuses on GLPG2737 as an add-on treatment in F508del homozygous patients on stable treatment with Orkambi and a second study is evaluating GLPG2737 as a triple combination with GLPG2222 and GLPG2451. These studies will help in understanding the translation of the *in vitro* observed effects to patients.

## Author Contributions

GdW and KC: conceptualization. MG, SM, KV, MJ, A-SW, T-CH, TC, MP, SVdP, ND, MC, PS, LN, and AS: investigation. KC: writing original draft. GdW, MG, and KC: writing – review and editing. MG and KC: visualization.

## Conflict of Interest Statement

GdW, MG, SM, KV, MJ, A-SW, TC, MP, SVdP, ND, PS, LN, and KC are employees of Galapagos and may own stock options in that company. AS and MC are employees of AbbVie and may own stock in that company. The remaining author declares that the research was conducted in the absence of any commercial or financial relationships that could be construed as a potential conflict of interest.

## Footnotes

1 www.genet.sickkids.on.ca

2 www.CFTR2.org
