# Supplementary material for: Identification of GLPG/ABBV-2737, a Novel Class of Corrector, Which Exerts Functional Synergy With Other CFTR Modulators
Source: Front Pharmacol. 2019 May 9;10:514. doi: 10.3389/fphar.2019.00514 (PMC6521598; doi:10.3389/fphar.2019.00514)
Supplement: Supplementary file 1 [file Data_Sheet_1.pdf]

Supplementary Material

Identification of GLPG/ABBV-2737, a novel class of corrector, which exerts functional synergy with other CFTR modulators

Gert de Wilde, Maarten Gees, Sara Musch, Katleen Verdonck, Mia Jans, Anne-Sophie Wesse, Ashvani K. Singh, Tzyh-Chang Hwang, Thierry Christophe, Mathieu Pizzonero, Steven Van der Plas, Nicolas Desroy, Marlon Cowart, Pieter Stouten, Luc Nelles, Katja Conrath*

*** Correspondence:** Corresponding Author: katja.conrath@glpg.com

1 Supplementary Figures

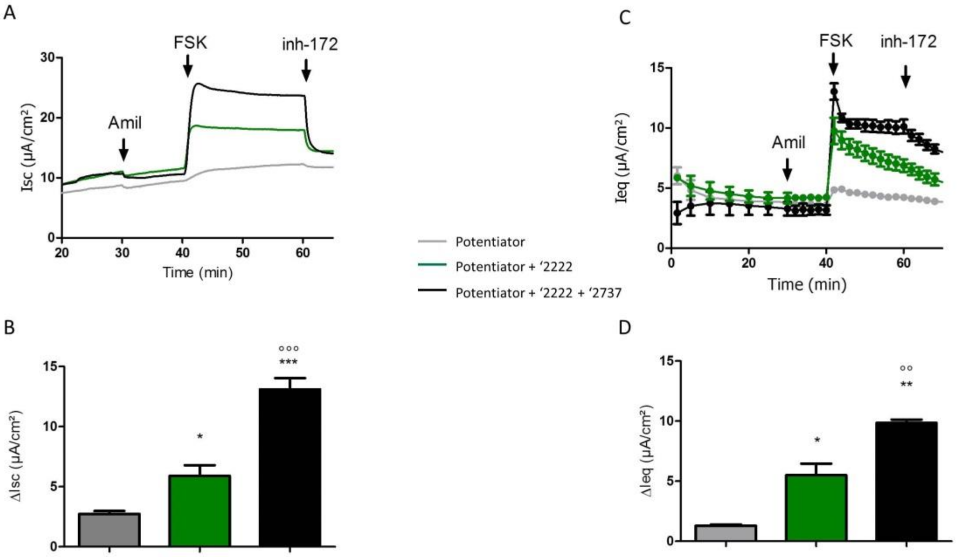

Supplementary Figure 1.

Evaluation of GLPG2737 corrector activity in Ussing chamber and TECC on F508del/F508del HBE cells (donor BCF714). **A.** Average traces of Ussing chamber data of cells treated with either 2 µM potentiator (grey); 2 µM potentiator + 0.15 µM GLPG2222 (green) or 2 µM potentiator + 0.15 µM GLPG2222 + 1µM GLPG2737 (black). **B.** Changes in short circuit current after stimulation with forskolin for the experiment shown in A (n= 3 for each condition) **C.** traces of TECC data of cells treated with either Potentiator (grey); Potentiator + GLPG2222 (green) or Potentiator + GLPG2222 + GLPG2737 (black). (n= 3 for each condition) **D.** Area under Curve calculations. Potentiator used was GLPG2451. p values are denoted with * compared to DMSO and with ° compared to GLPG2222 + potentiator (one symbol meaning $p < 0.05$, 2 $p < 0.01$ and 3 < 0.0001)

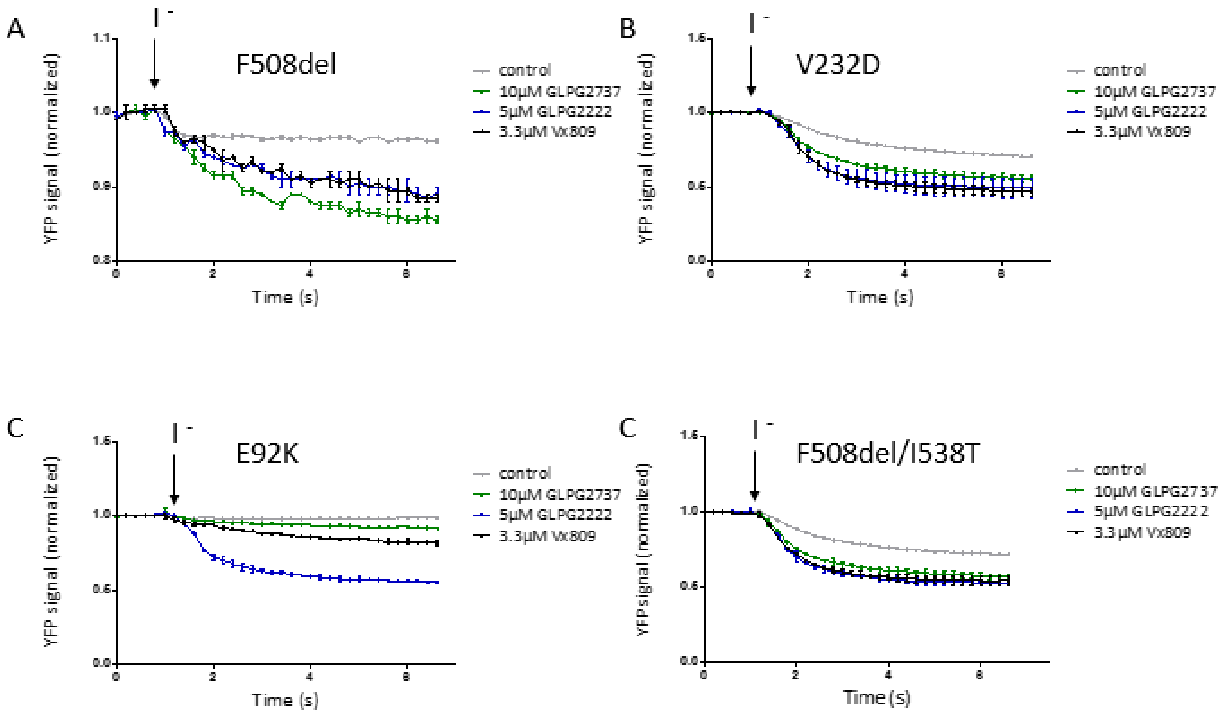

Supplementary Figure 2: YHA assay using CFTR mutant overexpression in HEK293 cells. Cells were incubated for 24 hours with either DMSO (grey); 10 µM GLPG2737 (green); 5 µM GLPG2222 (blue); 3.3 µM VX-809 (black). After washing, cells were triggered with forskolin (50 µM for F508del (A), V232D (B), E92K (C) and F508del/I539T (D)) and 0.5 µM potentiator and YFP fluorescence was measured. The YFP fluorescence was recorded during 7 s, starting directly before iodide injection. The CFTR channel function was expressed using the formula (1-(YFP fluorescence 7 s after iodide addition (F) / YFP fluorescence before iodide addition (F0))

	EC_{50} (nM)	normalised efficacy (%)	n
Donor1	14.1 ± 4.5	171 ± 27	3
Donor2	27.8 ± 26.9	208 ± 40	2
Donor3	18.9 ± 7.4	172 ± 11	2
Donor4	5.9	187	1

Supplementary Table 1: Dose dependent effect of GLPG2737 on F508del CFTR function. F508del/F508del HBE cells for different donors were incubated for 24 hours with a dose response of GLPG2737 in combination with 0.15 µM GLPG2222 and 1.5 µM GLPG3067. The potency (EC_{50}) and normalized efficacy (with response of GLPG2222 and GLPG3067 set as 100%) in each donor are shown.
